# Supplementary material for: Antagonistic control of intracellular signals by EpOMEs in hemocytes induced by PGE2 and their chemical modification for a potent insecticide
Source: PLoS One. 2025 May 7;20(5):e0320488. doi: 10.1371/journal.pone.0320488 (PMC12057851; doi:10.1371/journal.pone.0320488)
Supplement: S1 Table — (DOCX) [file pone.0320488.s001.docx]

**S1 Table.** Toxicity (LD50 ng/larva) of EpOME alkoxides against last instar larvae of *P. xylostella* (Px), *M. vitrata* (Mv) and *S. exigua* (Se) at hemocoelic injection

| **Compound** | **LD50 (ng/larva) ± SD** | | |
| --- | --- | --- | --- |
|  | Px | Mv | Se |
| EpOME | 283.00 ± 105.44 | 11958.88 ± 11642.37 | 23452.67 ± 6994.72 |
| A841 | 19.28 ± 9.91 | 139.06 ± 39.06 | 7244.64 ± 5268.27 |
| PD23 | 43.54 ± 29.56 | 70.10 ± 29.89 | 1738.84 ± 992.07 |
| PD28 | 5.50 ± 3.94 | 19.43 ± 13.27 | 178.64 ± 144.00 |
| AS46 | 30.88 ± 10.50 | 98.62 ± 29.97 | 704.69 ± 697.15 |
| AS56 | 0.75 ± 0.37 | 12.25 ± 4.83 | 9.83 ± 5.91 |
