# Supplementary material for: Antagonistic control of intracellular signals by EpOMEs in hemocytes induced by PGE2 and their chemical modification for a potent insecticide
Source: PLoS One. 2025 May 7;20(5):e0320488. doi: 10.1371/journal.pone.0320488 (PMC12057851; doi:10.1371/journal.pone.0320488)
Supplement: S2 Table — (DOCX) [file pone.0320488.s002.docx]

**S2 Table.** Toxicity (LC50 ppm) of EpOME alkoxides against last instar larvae of *P. xylostella* (Px), *M. vitrata* (Mv) and *S. exigua* (Se) at feeding assay

| **Compound** | **LC50 (ppm) ± SD** | | |
| --- | --- | --- | --- |
|  | Px | Mv | Se |
| EpOME | 15275.33 ± 2099.12 | 35243 ± 0.00 | 44162.00 ± 7671.55 |
| A841 | 911.64 ± 205.70 | 285.54 ± 74.11 | 1312.78 ± 582.31 |
| PD23 | 2142 ± 705.24 | 1029.96 ± 490.28 | 518.01 ± 217.13 |
| PD28 | 724.63 ± 117.80 | 147.61 ± 56.40 | 180.94 ± 69.55 |
| AS46 | 740.16 ± 555.05 | 418.26 ± 37.18 | 784.93 ± 173.40 |
| AS56 | 8.66 ± 0.66 | 57.57 ± 17.32 | 55.43 ± 33.66 |
