# Supplementary material for: Antagonistic control of intracellular signals by EpOMEs in hemocytes induced by PGE2 and their chemical modification for a potent insecticide
Source: PLoS One. 2025 May 7;20(5):e0320488. doi: 10.1371/journal.pone.0320488 (PMC12057851; doi:10.1371/journal.pone.0320488)
Supplement: S3 Table — (DOCX) [file pone.0320488.s003.docx]

**S3 Table.** Median lethal time (LT50) in days for last instar of *S. exigua* at 100 ppm of *B.*

*thuringiensis* (Bt), 10 ppm of AS56 and combination of Bt (100 ppm) and AS56 (10 ppm)

| **LT50 (day) ± SD** | | | |
| --- | --- | --- | --- |
|  | Bt | AS56 | Bt + AS56 |
| *S. exigua* | 6.39 ± 1.11 | 5.07 ± 0.41 | 3.01 ± 0.16 |
