# Supplementary material for: Antagonistic control of intracellular signals by EpOMEs in hemocytes induced by PGE2 and their chemical modification for a potent insecticide
Source: PLoS One. 2025 May 7;20(5):e0320488. doi: 10.1371/journal.pone.0320488 (PMC12057851; doi:10.1371/journal.pone.0320488)
Supplement: S1 Document — (DOCX) [file pone.0320488.s004.docx]

**S1 Document.** Chemical synthesis of EpOME alkoxides

## General information

All commercially available reagents and solvents were used in the form they were supplied without further purification. The stated yields are based on isolated material. Thin layer chromatography was performed on silica gel 60 F254 aluminum-backed plates fabricated by Merck. Flash column chromatography was performed on silica gel 60 (40 - 63 μm) fabricated by Merck. NMR spectra were recorded on a Bruker AVneo400, Bruker AVIII HD 400, or Bruker AVI600 spectrometer at 400 MHz or 600 MHz for 1H NMR and at 101 MHz or 151 MHz for 13C NMR. Coupling constants (J) are reported in hertz and chemical shifts are reported in parts per million (δ) relative to the central residual protium solvent resonance in 1H NMR (CDCl3 = δ 7.26) and the central carbon solvent resonance in 13C NMR (CDCl3 = δ 77.00 ppm). High- resolution mass spectra were recorded on a maXis Ⅱ ETD mass spectrometer using electrospray ionization as the ionization source. Optical rotations were measured using a 0.2 mL cell with a 0.1 dm path length on an Anton Paar MCP 100 polarimeter. HPLC-UV analyses were performed using an Agilent 1260 Series instrument with a ChiralPak AD-H column (ID 4.6 × 250 mm, particle size 5 μm), applying the conditions stated.

# Experimental Details

##
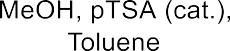
Methyl 9-bromononanoate


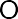

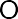


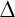
To a stirred solution of 9-bromononanoic acid (2.371 g, 10.00 mmol, 1.00 eq.) in toluene (30 mL) was added MeOH (15.0 mL, 370 mmol, 37.0 eq.) and catalytic amounts of *p*- toluenesulfonic acid (9 mg). The reaction mixture was heated to reflux, and stirred overnight with azeotropic removal of water. The solution was then cooled to room temperature, and the organic layer was washed with NaHCO3 (aq., 2 x 30 mL), dried over anhydrous MgSO4 and concentrated under reduced pressure. This afforded the title compound as a colourless oil (2.391 g, 9.52 mmol, 95%) which was used directly in the next step without further purification. **^1^H NMR (400 MHz, CDCl3):** δ 3.65 (s, 3H), 3.39 (t, *J* = 6.9 Hz, 2H), 2.29 (t, *J* = 7.5 Hz, 2H),

1.87-1.80 (m, 2H), 1.64-1.57 (m, 2H), 1.45-1.37 (m, 2H), 1.32-1.28 (m, 6H) **^13^C NMR (101**

**MHz, CDCl3):** δ 174.2, 51.4, 34.0, 33.9, 32.7, 29.0, 29.0, 28.9, 28.5, 28.0, 24.8. The spectral

data were in agreement with those reported in literature for methyl 9-bromononanoate^1^.

**
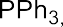
(9-methoxy-9-oxononyl)triphenylphosphonium bromide**


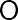


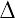
To a stirred solution of methyl 9-bromononanoate (2.239 g, 8.915 mmol, 1.00 eq.) in anhydrous acetonitrile (20 mL) was added triphenylphosphine (9.479 g, 36.14 mmol, 4.00 eq.) in one portion. The reaction mixture was refluxed overnight, and then cooled to room temperature. The solvent was removed *in vacuo*, and the crude residue was purified by silica gel flash chromatography (gradient eluent, 0-5% MeOH in DCM), which afforded the title product as a white semi-solid (4.497 g, 8.758 mmol, 98%). **^1^H NMR (400 MHz, CDCl3):** δ 7.88-7.76 (m, 9H), 7.79-7.76 (m, 6H), 3.85-3.78 (m, 2H), 3.63 (s, 3H), 2.25 (t, *J* = 7.3 Hz, 2H), 1.63-1.50 (m, 6H), 1.27-1.20 (m, 6H); **^13^C NMR (400 MHz, CDCl3):** δ 174.3, 134.9 (*JCP* = 3.2 Hz), 133.7 (*JCP* = 10.2 Hz), 130.4 (*JCP* = 12.6 Hz), 118.4 (*JCP* = 85.7 Hz), 51.4, 33.9, 30.3 (*JCP* = 15.8 Hz), 28.9 (*JCP* = 1.05 Hz), 28.9, 28.7, 24.7, 22.6 (*JCP* = 4.65 Hz). 22.3 (*JCP* = 49.6 Hz). The spectral data were in agreement with those reported in literature for (9-methoxy-9- oxononyl)triphenylphosphonium bromide^1^.

## Grignard Reaction - General Procedure

To a stirred solution of the Grignard reagent (8.0 mmol, 2.0 eq.) in anhydrous THF (8.0 mL) kept in an NaCl:ice cooling bath, the aldehyde (4.0 mmol, 1.0 eq.) was added dropwise. The reaction mixture was stirred for 10 minutes at this temperature, after which time TLC (KMnO4- stain) showed full conversion of the starting material. The reaction was quenched by addition of H2O (1 mL), and the resulting mixture was further diluted in H2O:Et2O (1:1, 30 mL). The layers were separated, and the aqueous phase was extracted using Et2O (2 x 15 mL). The combined organic layers were washed with H2O (15 mL) and brine (15 mL), dried over anhydrous MgSO4, and concentrated under reduced pressure to furnish the desired product. The residue was used directly in the next step without further purification.

### (±)-Non-1-en-4-ol:


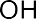


(±)-Non-1-en-4-ol was prepared according to the general procedure using allylmagnesium chloride (2.0 M in THF, 20.0 mL, 40.0 mmol) and hexanal (2.45 mL, 20.0 mmol) as the starting materials. **Yield:** 1.80 g (12.7 mmol, 63%), colourless oil; **R*f* (15% EtOAc in heptane)** = 0.26;

**^1^H NMR (400 MHz, CDCl3):** δ 5.83- 5.79 (m, 1H), 5.15-5.10 (m, 2H), 3.65-3.62 (m, 1H), 2.33-

2.26 (m, 1H), 2.17- 2.09 (m, 1H), 1.64 (s, 1H), 1.49- 1.25 (m, 8H), 0.89 (t, *J* = 6.82 Hz, 3H);

**^13^C NMR (101 MHz, CDCl3):** δ 134.9, 118.0, 70.7, 41.9, 36.7, 31.8, 25.3, 22.6, 14.0; **IR (ATR,**

**cm^-1^)**; 3348 (OH), 3081(=CH), 1645(C=C). The spectral data were in agreement with those reported in literature for non-1-en-4-ol^2^.

### (±)-Non-1-en-3-ol:


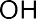


(±)-Non-1-en-3-ol was prepared according to the general procedure using vinylmagnesium bromide (1.0 M in THF, 30.0 mL, 30.0 mmol) and heptanal (2.1 mL, 15.0 mmol) as the starting materials. **Yield:** 2.12 g (14.9 mmol, 99 %), pale yellow oil; **Rf (30% EtOAc in heptane)** = 0.30; **^1^H NMR (400 MHz, CDCl3):** δ 5.90- 5.82 (m, 1H), 5.23- 5.18 (m, 1H), 5.11- 5.07 (m, 1H), 4.11-

4.06 (m, 1H), 1.59- 1.25 (m, 11H), 0.89-0.86 (m, 3H); **^13^C NMR (101 MHz, CDCl3):** δ 141.3,

114.4, 73.2, 37.0, 31.7, 29.5, 25.2, 22.5, 14.0; **IR (ATR, cm^-1^):** 3361 (O-H), 1645 (C=C). The

spectral data were in agreement with those reported in literature for (3*S*)-non-1-en-3-ol^3^.

### (±)-Dec-1-en-4-ol:


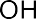


(±)-Dec-1-en-4-ol was prepared according to the general procedure using allylmagnesium chloride (2.0 M in THF, 4.0 mL, 8.0 mmol) and heptanal (0.56 mL, 4.0 mmol) as the starting materials. **Yield:** 588 mg (3.76 mmol, 94%), colourless oil; **R*f* (15% EtOAc in heptane) =** 0.20;

**^1^H NMR (400 MHz, CDCl3):** δ 5.86-5.78 (m, 1H), 5.16-5.10 (m, 2H), 3.68-3.61 (m, 1H), 2.34-

2.27 (m, 1H), 2.17-2.09 (m, 1H), 1.61-1.22 (m, 11H), 0.89 (t, *J* = 6.98 Hz), 3H); **^13^C NMR (101**

**MHz, CDCl3):** δ 134.9, 118.0, 70.7, 41.9, 36.8, 31.8, 29.3, 25.6, 22.6, 14.1. The spectral data

were in agreement with those reported in literature for *(S)-* and *(R)-*dec-1-en-4-ol^4,^ ^5^.

## Brown Allylation – General Procedure

Heptanal (0.55 mL, 3.9 mmol, 1.0 eq.) was dissolved in anhydrous Et2O (16 mL), and the resulting mixture was cooled to -100 °C on a dry ice/Et2O cooling bath. To this solution, (±)- Ipc2BAllyl borane solution (0.5 M in pentane/Et2O, 8.0 mL, 4.0 mmol, 1.0 eq.) was added dropwise *via* a pre-cooled syringe at -78 °C over the course of 1 hour. Stirring was continued at -100 °C for 2 hours, before MeOH (0.81 mL) was added, and the cooling bath was removed. A mixture of THF/H2O (1:1, 16 mL) was then added to the reaction flask, followed by addition of NaBO3•4H2O (2.42 g, 15.7 mmol, 4.00 eq.). The resulting suspension was stirred at ambient temperature overnight, after which time the mixture was further diluted with H2O (16 mL), and the phases were separated. The aqueous layer was extracted with Et2O (3 x 20 mL), the combined organic layers were dried over anhydrous MgSO4 and concentrated under reduced pressure. The crude residue was purified by silica gel flash chromatography (gradient elution, 0-15% EtOAc in *n-*heptane, R*f* = 0.20, KMnO4-stain) which afforded the desired compound as a colourless oil.

### *(S)*-4-dec-1-enol


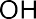


(*S)-*4-dec-1-enol was prepared according to the general procedure using (+)-Ipc2BAllylborane (0.5 M solution in pentane/Et2O, 8 mL, 4 mmol) and heptanal (0.55 mL, 3.9 mmol) as the starting materials. **Yield:** 460 mg (2.94 mmol, 76%). (*S*)-4-dec-1-enol gave rise to identical ^1^H and ^13^C NMR spectra as the racemate. The enantiomeric excess (95%) was determined by derivatisation of the alcohol with 2-napthoyl chloride and subsequent HPLC-UV analysis. [𝜶]^𝟐𝟓^ = -5.9 ° (CHCl3, c = 1.0)

𝑫

### *(R)*-4-dec-1-enol


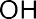


*(R)-*4-dec-1-enol was prepared according to the general procedure using (-)-Ipc2BAllylborane (0.5 M solution in pentane/Et2O, 10 mL, 5 mmol) and heptanal (0.70 mL, 5 mmol) as the starting materials. **Yield:** 625 mg (4.00 mmol, 80%), colourless oil. *(R)*-4-dec-1-enol produced identical ^1^H and ^13^C NMR spectra as its enantiomer. The enantiomeric excess (96%) was determined by derivatisation of the alcohol with 2-napthoyl chloride and subsequent HPLC-UV analysis. [𝜶]^𝟐𝟓^ = + 10 ° (CHCl3, c = 1.0)

𝑫

## Dec-1-en-4-yl 2-naphthoate

To an ice-cold, stirred solution of dec-1-en-4-ol in DCM (0.21 M, 1.00 eq.), Et3N (3.00 eq.), DMAP (10.0 mol%), and then 2-napthoyl chloride (1.20 eq.) were added successively. The reaction mixture was heated to ambient temperature overnight, and then concentrated under reduced pressure. The concentrate was suspended in *n-*heptane:NaH2PO4 (aq.) (1:1), and stirred for 5 minutes. Subsequently, the layers were separated, and the aqueous phase was extracted with *n*-heptane (3 x). The combined extracts were dried over anhydrous MgSO4 and concentrated under reduced pressure. The residue was purified by silica gel flash column chromatography (gradient elution, 0-10% EtOAc in *n*-heptane) which afforded the naphtalate as a viscous oil.

### (±)-dec-1-en-4-yl-2-napthtoate

**Yield:** 38 mg (0.12 mmol, 80%); **R*f* (10% EtOAc in heptane, visualised by UV and KMnO4- stain) =** 0.43; **^1^H NMR (400 MHz, CDCl3):** δ 8.61-8.60 (m, 1H), 8.08-8.06 (dd, *J =* 1.71, *J =* 8.63 Hz, 1H), 7.98-7.96 (m, 1H), 7.89-7.87 (d, *J* = 8.41, 2H), 7.61-7.52 (m, 2H), 5.93-5.82 (m, 1H), 5.28-5.22 (m, 1H), 5.17-5.06 (m, 2H), 2.52-2.47 (m, 2H), 1.82-1.67 (m, 2H), 1.48-1.26 (m, 8H), 0.87 (m, 3H) **^13^C NMR (101 MHz, CDCl3):** δ 166.4, 135.5, 133.8, 132.5, 130.9, 129.4, 128.1, 128.1, 128.0, 127.8, 126.6, 125.3, 117.8, 74.2, 38.8, 33.8, 31.7, 29.2, 25.4, 22.6, 14.1; **HRMS (ESI):** *m/*z calculated for C21H26O2Na [M + Na]^+^: 333.1825, found: 333.1824. The enantiomeric excess (50%) was determined by HPLC-UV (Chiralpak AD-H, *i*-PrOH/hexane 2:98, 1.0 mL/min), 2:98, 1.0 mL/min), *tR* (1) = 4.136 min, *tR* (2) = 4.362 min

### *(S)*-dec-1-en-4-yl-2-naphtoate

**Yield:** 32 mg (0.10 mmol, 89%). The (*S*)-enantiomer gave rise to identical ^1^H and ^13^C NMR spectra as the racemate. The enantiomeric excess (95%) was determined by HPLC-UV (Chiralpak AD-H, i-PrOH/hexane, 2:98, 1.0 mL/min), *tR* (major) = 4.136 min, *tR* (minor) = 4.362 min. [𝜶]^𝟓^ = - 8.4 ° (CHCl3, c = 0.085)

𝑫

### *(R)*-dec-1-en-4-yl-2-naphtoate

**Yield:** 28 mg (0.090 mmol, 93%). The (*R*)-enantiomer gave rise to identical ^1^H and ^13^C NMR spectra as the racemate. The enantiomeric excess (96%) was determined by HPLC-UV (Chiralpak AD-H, i-PrOH/hexane, 2:98, 1.0 mL/min), *tR* (minor) = 4.139 min min, *tR* (major) = 4.368 min**.** [𝜶]^𝟓^ = + 12 ° (CHCl3, c = 0.091)

𝑫

## Williamson Ether Synthesis – General Procedure

NaH (60% dispersion in mineral oil, 728 mg, 18.2 mmol, 10.00 eq.) was measured out in a flame-dried two-neck round bottom flask, and washed with THF (2 x 15mL). The salt was suspended in THF (15 mL), before the alkenol (1.82 mmol, 1.00 eq.) and 1-bromopropane (2.48 mL, 27.3 mmol, 15.0 eq.) were added. The reaction mixture was heated to reflux and stirred overnight at elevated temperature. The suspension was cooled to room temperature and the reaction was quenched by addition of saturated aqueous NH4Cl (15 mL). The aqueous layer was extracted with Et2O (3 x 20 mL), and the combined organic layers were washed with water (20 mL) and brine (20 mL), dried over anhydrous Na2SO4, and concentrated under reduced pressure. The crude residue was purified by silica gel column flash chromatography (gradient elution, 0-4% EtOAc in *n*-heptane, KMnO4-stain), which afforded the desired ether.

### (±)-4-propoxynon-1-ene

(±)-4-propoxynon-1-ene was prepared according to the general procedure using (±)-non-1-en- 4-ol (1.45 g, 10.0 mmol) as the starting alcohol. **Yield**: 1.17 g (6.35 mmol, 62 %), colorless oil; **R*f* (1.5% EtOAc in heptane, KMnO4-stain) =** 0.27; **^1^H NMR (400 MHz, CDCl3):** δ 5.83-5.77 (m, 1H), 5.08-5.00 (m, 2H), 3.46-3.42 (m, 1H), 3.37-3.31 (m, 1H), 3.29-3.23 (m, 1H), 2.30-2.19 (m, 2H), 1.61-1.23 (m, 10H), 0.93-0.86 (m, 6H). **^13^C NMR** (101 MHz, CDCl3): δ 135.3, 116.5, 79.0, 70.8, 38.5, 33.9, 32.0, 25.1, 23.3, 22.6, 14.0, 10.7. **IR** (ATR, cm^-1^): 1089 (CO). **HRMS (ESI)** *m/z* calculated C12H24NaO [M+Na]^+^**:** 207.1719 m/z, found: 207.1718 m/z**.**

### (±)-3-propoxynon-1-ene

(±)-3-propoxynon-1-ene was prepared according to the general procedure using (±)-non-1-en- 3-ol (2.10 g, 14.8 mmol) as the starting alcohol. **Yield**: 1.88 g (10.2 mmol, 69%), pale yellow oil. **R*f*** (**2% EtOAc in heptane, KMnO4-stain) =** 0.27; **^1^H NMR (400 MHz, CDCl3)**: δ 5.71-5.62 (m, 1H), 5.17-5.12 (m, 2H), 3.60-3.55 (m, 1H), 3.47-3.41 (m, 1H), 3.23-3.18 (m, 1H), 1.64-1.23 (m, 12H), 0.95-0.81 (m, 6H); **^13^C NMR (101 MHz, CDCl3)**: δ 139.8, 116.0, 81.3, 70.2, 35.5, 31.8, 29.2, 25.4, 23.1, 22.6, 14.1, 10.7; **IR** (ATR, cm^-1^): 1091(C-O); **HRMS (ESI);** *m/z* calculated C12H24NaO [M+Na]^+^: 207.1719, found: 207.1720**.**

### (*S*)-4-propoxydec-1-ene

**
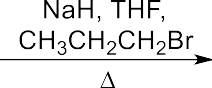
**


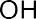

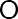


*(S)*-4-propoxydec-1-ene was prepared according to the general procedure using *(S)*-4-dec-1- enol (284 mg, 1.82 mmol) as the starting alcohol. **Yield:** 289 mg (1.46 mmol, 80 %); **R*f* (4% EtOAc in *n*-heptane)** *=* 0.27; **^1^H NMR (400 MHz, CDCl3):** δ 5.88-5.78 (m, 1 H), 5.09-5.01 (m, 2H). 3.46-3.41 (m, 1H), 3.37-3.31 (m, 1H), 3.29-3.23 (m, 1H), 2.29-2.21 (m, 2H), 1.61-1.21 (m, 12H), 0.94-0.86 (m, 6H); **^13^C NMR (101 MHz, CDCl3):** δ 135.4, 116.5, 79.0, 70.8, 38.5, 34.0, 31.9, 29.4, 25.4, 23.4, 22.6, 14.1, 10.7; [𝜶]^𝟓^ = - 12.6 ° (CHCl3, c = 0.05); **HRMS (ESI):** *m/z* calculated for C13H26ONa [M + Na]^+^: 221.1876, found: 221.1877

𝑫

### (*R*)-4-propoxydec-1-ene

**
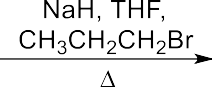
**


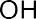

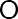


*(R)-*4-propoxydec-1-ene was prepared according to the general procedure using *(R)*-4-dec-1- enol (305 mg, 1.95 mmol) as the starting alcohol. **Yield:** 292 mg (1.47 mmol, 75%); *(R)*-4- propoxydec-1-ene gave rise to identical ^1^H and ^13^C NMR spectra as its enantiomer.

[𝜶]^𝟐𝟓^ = + 18 ° (CHCl3, c = 0.10)

𝑫

## Anti-Markovnikov Hydroboration – General Procedure

The alkene (5.43 mmol, 1.0 eq.) was measured out in an oven-dried round bottom flask and dissolved in anhydrous THF (50 mL). The stirred solution was cooled to 0 °C, before BH3•DMS (8.31 mL, 8.14 mmol, 1.0 M in THF, 1.5 eq.) was added in a dropwise manner. After 5 hours, aqueous NaOH (8.31 mL, 8.14 mmol, 1.0 M, 1.5 eq.) was added slowly to the ice-cold mixture, and stirring was continued for 1 hour. H2O2 (0.94 mL, 31.2 mmol, 5.7 eq) was then added to the mixture, and the reaction was stirred for 2 additional hours. The solution was diluted in H2O, and the aqueous layer was extracted using EtOAc (2 x 30 mL). The combined organic layers were washed with brine (1 x 25 mL), dried over anhydrous MgSO4, and concentrated under reduced pressure. The crude residue was purified by silica gel flash chromatography, which afforded the desired products as colourless oils.

### 4-propoxynonan-1-ol

**
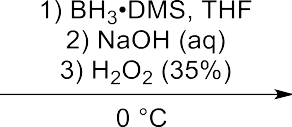
**


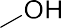


4-propoxynonan-1-ol was prepared according to the general procedure using 4-propoxynon- 1-ene (1.000 g, 5.43 mmol) as the starting alkene. **Yield**: 0.601 g (2.97 mmol, 55%); **R*f* (30% EtOAc in heptane, KMnO4-stain)** = 0.20; **^1^H NMR (400 MHz, CDCl3):** δ 3.63-3.59 (m, 2H), 3.41-3.32 (m, 2H), 3.28-3.22 (m, 1H), 2.52 (s, 1H), 1.69-1.22 (m, 14H), 0.92-0.85 (m, 6H); **^13^C NMR (101 MHz, CDCl3):** δ 79.4, 70.6, 63.0, 33.5, 32.0, 30.6, 28.6, 25.0, 23.3, 22.6, 14.0, 10.7; **IR (ATR, cm^-1^):** 3370 (O-H),1640 (C=C). **HRMS (ESI):** *m/z* calculated for C12H26Na1O2 [M+Na]^+^: 225.1825, found: 225.1825.

### 3-propoxynonan-1-ol

**
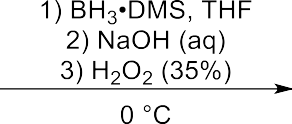
**

3-propoxynonan-1-ol was prepared according to the general procedure using 3-propoxynon- 1-ene (0.900 g, 4.88 mmol) as the starting alkene. **Yield**: 0.320 g (2.97 mmol, 33%); **R*f* (30% EtOAc in heptane, KMnO4-stain)** = 0.23; **^1^H NMR (400 MHz, CDCl3)**: δ 3.84-3.66 (m, 2H), 3.53-3.41 (m, 2H), 3.41-3.29 (m, 1H), 2.94-2.87 (m, 1H), 1.79-1.33 (m, 6H), 1.33-1.20 (m, 8H), 0.96-0.82 (m, 6H); **^13^C NMR (101 MHz, CDCl3)**: δ 79.6, 70.5, 61.0, 35.5, 33.3, 31.6, 29.3, 24.9, 23.2, 22.4, 13.9, 10.5. IR (ATR, cm^-1^):3381. **HRMS (ESI):** *m/z* calculated for C12H26Na1O2 [M+Na]^+^: 225.1825, found: 225.1828.

## Dess-Martin Oxidation – General Procedure

To a stirred solution of the alcohol (1.04 mmol, 1.00 eq.) in DCM (8 mL) was added NaHCO3 (180 mg, 2.08 mmol, 2.00 eq.) and DMP (710 mg, 1.66 mmol, 1.6 eq.). The reaction was stirred overnight, and then quenched by addition of aqueous Na2S2O3 (10 mL). The layers were separated, and the aqueous phase was extracted with DCM (3 x 5 mL). The combined extracts were dried over anhydrous MgSO4 and concentrated under reduced pressure. The crude residue was purified by silica gel flash chromatography, furnishing the desired aldehyde.

### (±)-4-propoxynonanal

**
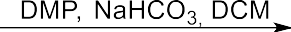
**


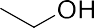

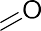


(±)-4-propoxynonanal was prepared using 4-propoxynonan-1-ol (211 mg, 1.04 mmol) as the starting alcohol. **Yield**: 190 mg (0.949 mmol, 91%). **R*f* (30% EtOAc in heptane, KMnO4-stain)**

= 0.32; **^1^H NMR (400 MHz, CDCl3):** δ 9.76 (t, *J* = 1.8 Hz, 1H), 3.42-3.34 (m, 1H), 3.31-3.26 (m,

1H), 3.26-3.19 (m,1H), 2.49 (td, *J* = 7.2, 1.81 Hz, 2H), 1.90-1.80 (m, 1H), 17.9-18.9 (m, 1H),

1.59-1.47 (m, 3H), 1.44-1.19 (m, 8H), 0.93-0.85 (m, 6H). **^13^C NMR** (101 MHz, CDCl3) δ 203.2,

78.9, 71.2, 40.6, 34.3, 32.5, 27.1, 25.5, 23.7, 23.1, 14.5, 11.2; **IR (ATR, cm^-1^):** 1727 (C=O).

**HRMS (ESI):** *m/z* calculated for C12H24NaO2 [M+Na]^+^: 223.1669, found: 223.1665

### (±)-3-propoxynonanal

**
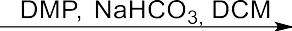
**


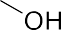


(±)-3-propoxynonanal was prepared using 3-propoxynonan-1-ol (303 mg, 1.50 mmol) as the starting alcohol. **Yield:** 180 mg (0.899 mmol, 60%); **R*f* (30% EtOAc in heptane, KMnO4-stain)**

= 0.44; **^1^H NMR (400 MHz, CDCl3):** δ 9.79 (m, 1H), 3.81-3.70 (m, 1H), 3.46-3.31 (m, 2H), 2.64-

2.44 (m, 2H), 1.63-1.15 (m, 14H), 0.94-0.83 (m, 6H). **^13^C NMR (101 MHz, CDCl3)**: δ 202.2,

75.0, 71.1, 48.5, 35.1, 31.9, 29.4, 25.3, 23.4, 22.7, 14.2, 10.8; **IR** (ATR, cm^-1^): 1727 (C=O);

**HRMS (ESI):** *m/z* calculated for C13H28NaO3 [M+Na]^+^: 255.1931, found: 255.1927.

## *m-*CPBA Epoxidation – General Procedure


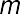


To an ice-cold, stirred solution of 4-propoxydec-1-ene (263 mg, 1.33 mmol) in DCM (25 mL) was added *m-*CPBA (895 mg, 3.99 mmol, ≤77% purity, 3.00 eq.) in one portion. The reaction mixture was heated to ambient temperature overnight, and then quenched by addition of aqueous Na2S2O3. The aqueous layer was extracted with DCM (3 x 20 mL), and the combined organic layers were washed with aqueous NaHCO3 (1 x 15 mL), water (1 x 15 mL), and brine (1 x 15 mL), dried over anhydrous MgSO4, and concentrated under reduced pressure. The crude residue was purified by silica gel column flash chromatography (10% EtOAc in *n-* heptane, CAM-stain, R*f* = 0.10), which afforded the title product as a mixture of diastereomers.

### 2-((*S*)-2-propoxyoctyl)oxirane

2-(*(S)*-propoxyoctyl)oxirane was prepared using *(S)*-4-propoxydec-1-ene (264 mg, 1.33 mmol) as the starting alkene. **Yield:** 222 mg (1.04 mmol, 78%); **^1^H NMR (400 MHz, CDCl3):** δ 3.49-3.36 (m, 3H), 3.09-3.00 (m, 1H), 2.81-2.75 (m, 1H), 2.50-2.46 (m, 1H), 1.80-1.25 (m, 14H), 0.94-0.86 (m, 6H). **^13^C NMR (101 MHz, CDCl3):** δ 77.3, 77.2, 71.3, 70.7, 50.0, 49.6, 47.6, 46.9, 37.9, 36.9, 34.7, 34.2, 31.8, 29.4, 29.4, 25.4, 25.2, 23.4, 23.3, 22.6, 14.1, 10.7; **HRMS (ESI):** *m/z* calculated for C13H26O2Na [M + Na]^+^: 1237.1825, found: 237.1821

### 2-((*R*)-2-propoxyoctyl)oxirane

2-(*(R)*-2-propoxyoctyl)oxirane was prepared using *(R)-*4-propoxydec-1-ene (203 mg, 1.02 mmol) as the starting alkene. **Yield:** 178 mg (0.830 mmol, 81%). The mixture of 2-((*R*)-2- propoxyoctyloxirane) diastereomers produced identical ^1^H and ^13^C NMR spectra as the 2-((*S*)- 2-propoxyoctyl)oxirane diastereomers.

## Epoxide Cleavage – General Procedure


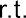
To a stirred solution of 2-(2-propoxyoctyl)oxirane (110 mg, 0.513 mmol, 1.00 eq.) in Et2O (6 mL) was added H5IO6 (167 mg, 0.733 mmol, 1.40 eq.). The reaction was stirred at room temperature for 1 hour, and then quenched by addition of aqueous NaHCO3. The phases were separated and the aqueous layer was extracted with Et2O (3 x 10 mL). The combined organic layers were washed with brine (1 x 10 mL), dried over anhydrous Na2SO4, and concentrated under reduced pressure. The crude residue was purified by silica gel flash column chromatography (10% EtOAc in *n-*heptane, KMnO4-stain, R*f* = 0.23).


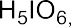

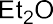


### (*S*)-3-propoxynonanal

**Yield:** 137 mg (0.68 mmol, 73%); [𝜶]*_D_*^𝟐𝟓^ = - 9.3 ° (CHCl3, c = 0.063)

The (*S*)-enantiomer produced identical ^1^H and ^13^C NMR spectra as the racemate.

### (*R*)-3-propoxynonanal

**Yield:** 116 mg (0.58 mmol, 79%) [𝜶]*_D_*^𝟐𝟓^ = + 5.2 ° (CHCl3, c = 0.023)

The (*R*)-enantiomer produced identical ^1^H and ^13^C NMR spectra as the racemate.

## Wittig Reaction – General Procedure

(9-methoxy-9-oxononyl)triphenylphosphonium bromide (174 mg, 0.34 mmol, 1.5 eq) was measured out in an oven-dried round bottom flask, azeotroped with 2-MeTHF (3 x 3 mL), and dissolved in a mixture of anhydrous THF (5.0 mL) and HMPA (1.5 mL). The reaction flask was evacuated for air, and purged under argon three times. The solution was cooled to – 78 °C on an acetone/dry ice cooling bath, before NAHMDS (0.52 mL, 0.31 mmol, 1.4 eq.) was added in a dropwise manner to furnish a deep orange color. After all of the base had been added, the reaction flask was raised above the cooling bath, and stirred at this temperature for 30 minutes. Subsequently, stirring was continued at – 78 °C for additional 30 minutes. Meanwhile, the aldehyde (45 mg, 0.22 mmol, 1.0 eq.) was azeotroped with 2-MeTHF (3 x 2 mL), and dissolved in anhydrous THF (1.5 mL). The resulting solution was purged under argon, then transferred to the flask containing the ylide in a dropwise manner *via* a pre-cooled syringe. Upon addition

of the aldehyde, the reaction mixture was stirred at – 78 °C for 1.5 hours. Then, the reaction mixture was heated to 0 °C and stirred at this temperature for 10 minutes, before the reaction was quenched by addition of aqueous NH4Cl (5.0 mL). After the suspension had stirred for 5 minutes, the layers were separated, and the aqueous phase was extracted using Et2O (3 x 10 mL). The combined organic layers were washed with brine (10 mL), dried over anhydrous Na2SO4, and concentrated under reduced pressure. The crude residue was purified by silica gel flash chromatography (2-5% EtOAc in heptane) to furnish the target compound as a colourless oil.

### (±)-Methyl (*Z*)-13-propoxyoctadec-9-enoate


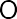


**Yield:** 90 mg (0.25 mmol, 25%); **R*f* (2% EtOAc in heptane) =** 0.15; **^1^H NMR (600 MHz, CDCl3):**

δ 5.37-5.32 (m, 2H), 3.66 (s, 3H), 3.40-3.33 (m, 2H), 3.23-3.19 (m, 1H), 2.31 (t, *J* = 7.69, 2H),

2.10-2.06 (m, 2H), 2.03-2.00 (m, 2H), 1.64-1.24 (m, 22H), 0.92 (t, *J =* 7.35 Hz, 3H), 0.89 (t, *J*

= 6.81 Hz, 3H); **^13^C NMR (151 MHz, CDCl3):** δ 174.3, 130.0, 129.6, 78.9. 70.6, 51.4, 34.1,

34.0, 32.1, 29.7, 29.2, 29.1, 27.2, 25.1, 24.9, 23.4, 23.2, 22.7, 14.8, 10.8; **HRMS (ESI):** m/z

calculated for C22H42O3 [M + Na]^+^: 377.3026, found 377.3024. The *(Z)*-double bond geometry was indicated by 2D ^1^H-^1^H NOESY spectroscopic analysis. **HRMS (ESI):** *m/z* calculated for C22H42O3Na [M + Na]; 377.3026, found: 377.3024

### (±)-Methyl *(Z)*-12-propoxyoctadec-9-enoate


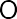


**Yield:** 60 mg (0.17 mmol, 22%); **R*f* (5% EtOAc in heptane) =** 0.15; **^1^H NMR (600 MHz, CDCl3):**

δ 5.42-5.40 (m, 2H), 3.66 (s, 3H), 3.46-3.43 (m, 1H), 3.35-3.31 (m, 1H), 3.24-3.21 (m, 1H),

2.30 (t, *J* = 7.64 Hz, 2H), 2.27-2.18 (m, 2H), 2.04-2.00 (m, 2H), 1.64-1.54 (m, 4H), 1.46-1.25

(m, 18H), 0.92 (t, *J* = 7.47, 3H), 0.88 (t, *J* = 7.08, 3H); **^13^C NMR (151 MHz, CDCl3):** δ 174.3,

131.5, 125.7, 79.5, 70.8, 51.4, 34.1, 34.1, 31.9, 31.8, 29.6, 29.5, 29.1, 27.4, 25.5, 24.9, 23.4,

22.6, 14.1, 10.7; The (*Z*)-double bond geometry was confirmed by 2D ^1^H-^1^H NOESY spectroscopic analysis. **HRMS (ESI):** *m/z* calculated for C22H42O3Na [M + Na]^+^; 377.3026, found: 377.3025.

### Methyl *(S,Z)*-12-propoxyoctadec-9-enoate


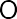

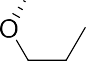


**Yield:** 68 mg (0.19 mmol, 35%) [𝜶]*_D_*^𝟐𝟓^ = - 20° (CHCl3, c = 0.063)

The (*S*)-enantiomer produced identical ^1^H and ^13^C NMR spectra as the racemate. The *(Z)*- double bond geometry was confirmed by 2D ^1^H-^1^H NOESY spectroscopic analysis.

### Methyl *(R,Z)*-12-propoxyoctadec-9-enoate


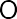


**Yield:** 49 mg (0.14 mmol, 63%) [𝜶]*_D_*^𝟐𝟓^ = + 6° (CHCl3, c = 0.020)

The (*R*)-enantiomer produced identical ^1^H and ^13^C NMR spectra as the racemate. The (*Z*)- double bond geometry was verified by 2D ^1^H-^1^H NOESY spectroscopic analysis.

# ^1^H NMR and ^13^C NMR Spectra of Compounds

### Methyl 9-bromononaoate


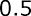

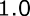

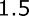

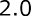

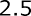

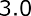

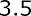

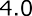

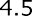

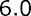

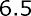

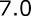

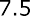

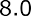

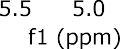

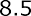

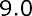

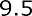

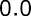

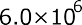

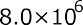

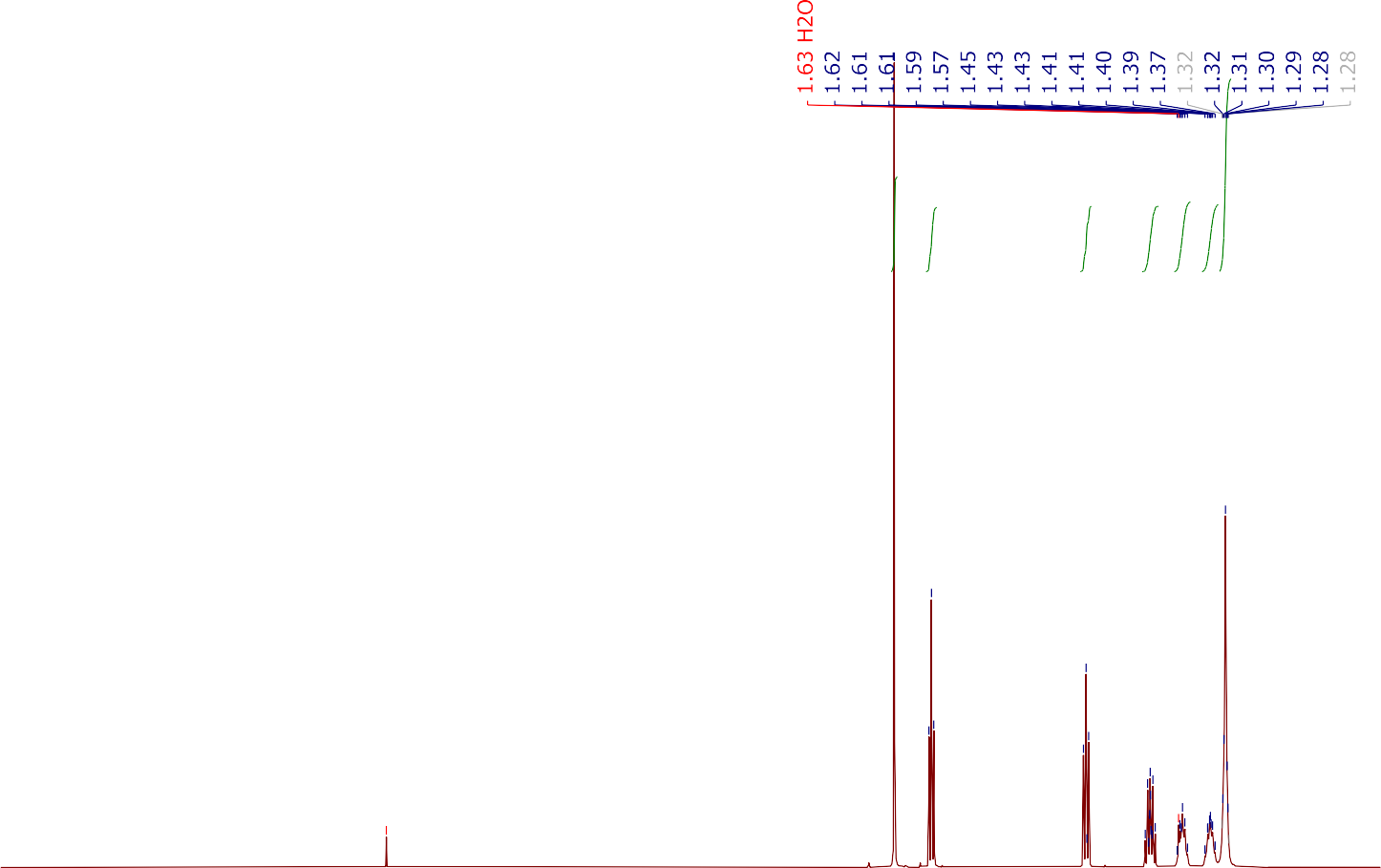

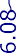

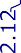

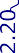

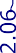

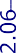

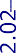

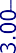

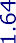

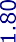

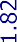

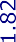

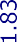

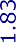

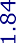

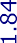

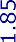

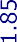

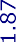

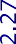

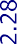

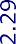

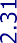

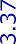

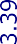

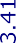

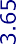

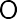


*This figure shows the ^1^H NMR (400 MHz, CDCl3) spectrum of methyl 9-bromononanoate.*


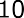

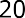

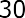

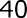

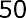

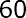

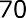

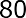

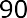

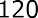

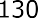

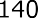

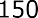

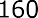

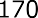

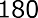


*This figure shows the 13C NMR (101 MHz, CDCl ) spectrum of methyl 9-bromononanoate.*

### (9-methoxy-9-oxononyl)triphenylphosphonium bromide

*This figure shows the 1H NMR (400 MHz, CDCl ) spectrum of (9-methoxy-9-oxononyl)triphenylphosphonium bromide.*

*This figure shows the ^13^C NMR (101 MHz, CDCl3) spectrum of (9-methoxy-9-oxononyl)triphenylphosphonium bromide.*

### Non-1-en-4-ol

*This figure shows the 1H NMR (400 MHz, CDCl ) spectrum of non-1-en-4-ol.*

*This figure shows the ^13^C NMR (101 MHz, CDCl3) spectrum non-1-en-4-ol.*

### Non-1-en-3-ol

*This figure shows the 1H NMR (400 MHz, CDCl ) spectrum of non-1-en-3-ol.*

*This figure shows the ^13^C NMR (101 MHz, CDCl3) spectrum of non-1-en-3-ol.*

### Dec-1-en-4-ol

*This figure shows the 1H NMR (400 MHz, CDCl ) spectrum of dec-1-en-4-ol. Both enantiomers produced identical spectra.*

*This figure shows the ^13^C NMR (101 MHz, CDCl3) spectrum dec-1-en-4-ol. Both enantiomers produced identical spectra.*

**Dec-1-en-4-yl-2-naphtoate**

*This figure shows the 1H NMR (400 MHz, CDCl ) spectrum of dec-1-en-4-yl-2-naphtoate. Both enantiomers produced identical spectra.*

*This figure shows the ^13^C NMR (101 MHz, CDCl3) spectrum of dec-1-en-4-yl-2-napthtoate. Both enantiomers produced identical spectra.*

### 4-propoxynon-1-ene

*This figure shows the 1H NMR (400 MHz, CDCl ) spectrum of 4-propoxynon-1-ene.*

*This figure shows the ^13^C NMR (101 MHz, CDCl3) spectrum of 4-propoxynon-1-ene.*

### 3-propoxynon-1-ene

*This figure shows the 1H NMR (400 MHz, CDCl ) spectrum of 3-propoxynon-1-ene.*

*This figure shows the ^13^C NMR (101 MHz, CDCl3) spectrum of 3-propoxynon-1-ene.*

### 4-propoxydec-1-ene

*This figure shows the 1H NMR (400 MHz, CDCl ) spectrum of 4-propoxydec-1-ene. Both enantiomers produced identical spectra.*

*This figure shows the ^13^C NMR (101 MHz, CDCl3) spectrum of 4-propoxydec-1-ene. Both enantiomers produced identical spectra.*

### 4-propoxynonan-1-ol

*This figure shows the 1H NMR (400 MHz, CDCl ) spectrum of 4-propoxynonan-1-ol.*

*This figure shows the ^13^C NMR (101 MHz, CDCl3) spectrum of 4-propoxynonan-1-ol.*

### 3-propoxynonan-1-ol

*This figure shows the 1H NMR (400 MHz, CDCl ) spectrum of 3-propoxynonan-1-ol.*

*This figure shows the ^13^C NMR (101 MHz, CDCl3) spectrum of 4-propoxynonan-1-ol.*

### 2-(2-propoxyoctyl)oxirane

*This figure shows the 1H NMR (400 MHz, CDCl ) spectrum of 2-(2-propoxyoctyl)oxirane*

*This figure shows the ^13^C NMR (101 MHz, CDCl3) spectrum of 2-(2-propoxyoctyl)oxirane.*

*This figure shows the DEPT-135 (101 MHz, CDCl3) spectrum of 2-(2-propoxyoctyl)oxirane.*

### 4-propoxynonanal

*This figure shows the 1H NMR (400 MHz, CDCl ) spectrum of 4-propoxynonanal.*

*This figure shows the ^13^C NMR (101 MHz, CDCl3) spectrum of 4-propoxynonanal.*

**3-propoxynonanal**

*This figure shows the 1H NMR (400 MHz, CDCl ) spectrum of 3-propoxynonanal. The enantiomers produced identical spectra.*

*This figure shows the ^13^C NMR (101 MHz, CDCl3) spectrum of 3-propoxynonanal. The* enantiomers produced identical spectra.

### Methyl *(Z)*-13-propoxyoctadec-9-enoate

*This figure shows the 1H NMR (600 MHz, CDCl ) spectrum of methyl (Z)-13-propoxyoctadec-9-enoate.*

*This figure shows the ^13^C NMR (151 MHz, CDCl3) spectrum of methyl (Z)-13-propoxyoctadec-9-enoate.*

*This figure shows the 1H-^1^H COSY (600 MHz, CDCl ) spectrum of methyl (Z)-13-propoxyoctadec-9-enoate.*

*This figure shows the 1H-^1^H TOCSY (600 MHz, CDCl ) spectrum of methyl (Z)-13-propoxyoctadec-9-enoate.*

*This figure shows the 1H-^1^H NOESY (600 MHz, CDCl ) spectrum of methyl (Z)-13-propoxyoctadec-9-enoate.*

*This figure shows the 1H-^13^C HSQC (600 MHz, CDCl ) spectrum of methyl (Z)-13-propoxyoctadec-9-enoate.*

*This figure shows the 1H-^13^C HMBC (600 MHz, CDCl ) spectrum of methyl (Z)-13-propoxyoctadec-9-enoate.*

### Methyl *(Z)*-12-propoxyoctadec-9-enoate

*This figure shows the 1H NMR (600 MHz, CDCl ) spectrum of methyl (Z)-12-propoxyoctadec-9-enoate. Both enantiomers gave rise to identical*

*spectra.*

*This figure shows the ^13^C NMR (151 MHz, CDCl3) spectrum of methyl (Z)-12-propoxyoctadec-9-enoate. Both enantiomers gave rise to*

*identical spectra.*

*This figure shows the 1H-^1^H COSY (600 MHz, CDCl ) spectrum of methyl (Z)-12-propoxyoctadec-9-enoate.*

*This figure shows the 1H-^1^H TOCSY (600 MHz, CDCl ) spectrum of methyl (Z)-12-propoxyoctadec-9-enoate.*

*This figure shows the 1H-^1^H NOESY (600 MHz, CDCl ) spectrum of methyl (Z)-12-propoxyoctadec-9-enoate.*

*This figure shows the 1H-^13^C HSQC (600 MHz, CDCl ) spectrum of methyl (Z)-12-propoxyoctadec-9-enoate.*

*This figure shows the 1H-^13^C HMBC (600 MHz, CDCl ) spectrum of methyl (Z)-12-propoxyoctadec-9-enoate.*

# Mass Spectra

### Methyl (*Z*)-13-propoxyoctadec-9-enoate

*This figure shows the HRMS-ESI spectrum of methyl (Z)-13-propoxyoctadec-9-enoate*

### Methyl (*Z)*-12-propoxyoctadec-9-enoate

*This figure shows the HRMS-ESI spectrum of methyl (Z)-13-propoxyoctadec-9-enoate.*

# HPLC-UV Chromatograms

### (±)-dec-1-en-4-yl-2-napthoate

*This figure shows the HPLC-UV chromatogram of the racemic mixture of dec-1-en-4-yl- 2-naphtoate.*

### *(S)*-dec-1-en-4-yl-2-naphtoate

*This figure shows the HPLC-UV chromatogram of (S)-dec-1-en-4-yl-2-naphtoate.*

### *(R)*-dec-1-en-4-yl-2-napthoate

*This figure shows the HPLC-UV chromatogram of (R)-dec-1-en-4-yl-2-naphtoate.*

# References

1. Jacquot, C.; McGinley, C. M.; Plata, E.; Holman, T. R.; van der Donk, W. A. Synthesis of 11-thialinoleic acid and 14-thialinoleic acid, inhibitors of soybean and human lipoxygenases. *Organic & Biomolecular Chemistry* **2008**, *6* (22), 4242-4252, [https://10.1039/B808003H](https://10.0.4.15/B808003H).
2. Wickel, S. M.; Citron, C. A.; Dickschat, J. S. 2H-Pyran-2-ones from Trichoderma viride and Trichoderma asperellum. *European Journal of Organic Chemistry* **2013**, *2013* (14), 2906-2913. DOI: <https://doi.org/10.1002/ejoc.201300049>.
3. Fenster, M. D. B.; Dake, G. R. An Asymmetric Formal Synthesis of Fasicularin. *Chemistry – A European Journal* **2005**, *11* (2), 639-649. DOI: <https://doi.org/10.1002/chem.200400749>.
4. Ortega, N.; Martín, V. S.; Martín, T. An Approach to Lauroxanes by Iterative Use of Co2(CO)6-Acetylenic Complexes. A Formal Synthesis of (+)-Laurencin. *The Journal of Organic Chemistry* **2010**, *75* (19), 6660-6672. DOI: [https://10.1021/jo101566x](https://10.0.3.253/jo101566x).
5. Zhang, Z.; Aubry, S.; Kishi, Y. Iterative Cr-Mediated Catalytic Asymmetric Allylation To Synthesize syn/syn- and anti/anti-1,3,5-Triols. *Organic Letters* **2008**, *10* (14), 3077-3080. DOI: [https://10.1021/ol801094e](https://10.0.3.253/ol801094e).
